# The Social Sources Adolescents Consult for Daily Life Choices: Variations in Age and Decision Domains

**DOI:** 10.1002/jad.70063

**Published:** 2025-10-12

**Authors:** Scarlett K. Slagter, Anna C.K. van Duijvenvoorde, Wouter van den Bos

**Affiliations:** ^1^ Department of Psychology University of Amsterdam Amsterdam the Netherlands; ^2^ Department of Developmental and Educational Psychology, Institute of Psychology Leiden University Leiden the Netherlands; ^3^ Amsterdam Brain and Cognition University of Amsterdam Amsterdam the Netherlands; ^4^ Max‐Planck‐Institut für Bildungsforschung Berlin Germany

**Keywords:** academic behaviour, adolescence, consulting, information‐seeking behaviour, prosocial behaviour, risky behaviour

## Abstract

**Introduction:**

Previous research has investigated the impact of peers on adolescents' decision‐making across various domains. However, adolescents are not just passive receivers of information; they actively seek advice from peers. Yet, there is limited understanding of whom adolescents turn to within their peer networks to guide their decisions.

**Methods:**

This study explored adolescents' preferences for seeking advice within their peer network when making decisions across different decision domains: risky, prosocial, and academic. Dutch youth (*N* = 748, ages 11–19) were presented with hypothetical scenarios and asked which classmates they preferred to consult. Peer nominations were used to examine the characteristics of consulted peers.

**Results:**

Primarily, adolescents seek information from their (best) friends—accounting for 70%–85% of cases—and peers they like and trust, with friends serving as the most important source of guidance across all domains. We also found that consulted peers were more likely to be perceived as cool, admirable, smart, influential, or as leaders, rather than lacking these characteristics. With increasing age, adolescents demonstrated an increased reliance on friends for prosocial and risky decisions and a greater bias for smart peers when making academic decisions.

**Conclusion:**

This study emphasises adolescents' active role in seeking advice from peers to inform their decisions related to risky‐, prosocial‐, and academic behaviour. Across all domains, adolescents prioritise guidance from (close) friends and peers they trust. Characteristics related to the social status of a peer, and perceived intelligence, also contribute to someone being consulted for advice. The type of peers adolescents prefer to consult appears to be more consistent across domains than highly domain‐specific. However, the increased bias for friends with age was absent for academic choices. Future studies should aim to better understand adolescents' motives for consulting certain peers and should investigate the extent to which a peer's knowledge and skills play a role. These insights are essential for evaluating the suitability of peers as information sources across various decision domains.

## 1 Introduction

Adolescents regularly face a variety of daily decisions, both minor and major, such as whether to accept an alcoholic drink, which subjects to choose at school, or how much to study for exams. They do not have to make these decisions on their own. Adolescents spend a considerable amount of time with their peers, particularly in school settings, where they are constantly interacting with classmates. When faced with a decision, adolescents can gather information by observing others or seeking advice from their peers (Agosto and Hughes‐Hassell 2006; Laland 2004). Based on this information, they can learn what is appropriate or which choice will likely lead to the most optimal outcome. Indeed, adolescents are not merely passive recipients of information. They actively seek information about their peers' actions before making their own decisions (Slagter et al. 2023; Slagter et al. 2023; Shenton and Dixon 2003). Adolescents particularly value this type of information for decisions related to health, school, social activities, fashion, and social relationships (Agosto and Hughes‐Hassell 2006). Considering adolescents' active role in seeking information from peers, a critical step is determining from whom to seek advice. Understanding which peers adolescents consider valuable sources for behavioural guidance is essential for comprehending the peer influence processes that occur in their daily lives. For example, these processes can lead to higher or lower tendencies to engage in risk‐taking (Albert, Chein and Steinberg 2013; Knoll et al. 2025), prosocial actions (Van Hoorn et al. 2016), school work, and can alter one's motivation for school (Raabe et al. 2019; Shin 2022; Shin and Ryan 2014). Understanding which peers adolescents consider as valuable sources for behavioural guidance is crucial for enhancing the positive aspects of social influence. However, there is a notable gap in the literature regarding the specific types of peers adolescents seek information from to guide their decisions.

Qualitative research on adolescents' information‐seeking behaviour identified family, friends and ‘other’ peers as one of the major sources that adolescents turn to for advice (Agosto and Hughes‐Hassell 2006; Shenton and Dixon 2003). However, these studies do not provide a detailed profile the peers that are a salient source, as they only assessed the role of friends, the general peer group and family. The social learning theory suggests that humans prefer to model and learn from individuals with a certain level of prestige, knowledge, or degree of similarity (Kendal et al. 2018; Laland 2004; Rendell et al. 2011). People are more likely to copy behaviours from prestigious individuals because these individuals are assumed to possess valuable knowledge or skills. Adolescents might seek to observe and imitate the behaviour of popular peers, believing their actions will learn them how to gain social status within the peer group (Dijkstra et al. 2010; Li and Wright 2014). Experimental research has shown that the impact of peers is moderated by the social status of the source (i.e. perceived popularity or those who are socially well‐connected; e.g. Choukas‐Bradley et al. 2015; Cohen and Prinstein 2006; Helms et al. 2014; Field et al. 2024). The social learning theory also assumes that people prefer to model individuals high on similarity, as shared experiences and challenges make their behaviours seem more relatable and relevant (Laland 2004). Adolescents may therefore prefer friends as sources of information, given that friends often share similar values, preferences and behaviours (Laursen 2017). The importance of friends is further supported by Social Identity Theory, which states that group membership is a key source of pride and self‐esteem. Adolescents are likely to align their choices with those of their friends to maintain a sense of belonging, as experiencing dissimilarity could disrupt their connection to this important social identity group (Spears 2021; Tajfel and Turner 1986). Many longitudinal and experimental studies have shown that friends are an important source of influence (e.g. Bot et al. 2005; Shin 2022; Field and Prinstein 2023). Lastly, the social learning theory suggest that people might also copy individuals who are competent or skilled in specific domains. While previous studies have shown that closeness and social status moderate the influence that peers have on adolescent's behaviour, it remains unclear whether adolescents also use these characteristics as criteria when selecting sources for advice.

In a recent experimental study we addressed this gap using a social sampling paradigm for risky decisions (Slagter et al. 2023). Here, adolescents were able to sample their peer's choices before making a risky decision. Results showed that ‘friendship’, ‘trustworthiness’ and ‘smartness’ were important predictive characteristics of consulted peers. Additionally, characteristics related to social status, such as being admirable and cool, increased the likelihood of being selected as a social source. However, we did not find evidence that adolescents preferred peers perceived as ‘popular’ to guide their decisions. Although this study provided a better understanding of which peers adolescents consult, it focussed solely on risky choices within an experimental paradigm. Whether these results replicate and generalise to decision‐making in daily life, across a variety of domains, should be investigated.

Adolescents' preference for specific sources may vary depending on the decision domain. From an early age, children already perceive adults as a better source for questions related to food, while they prefer to ask children for toy‐related questions (VanderBorght and Jaswal 2009). Another study by Sebald (1986) showed that adolescents tend to consult their parents mainly for decisions related to school, finances, and future career planning, while they seek advice from peers mainly for decisions concerning their social lives, such as dating, clothing, and alcohol consumption. This study suggests that adolescents' preference for social sources varies by decision domain, as the relevance or suitability of the source may be considered in each specific context. However, this study only made a distinction between parents and peers but did not reveal information about the types of peers that matter. Next to variations in source selection based on the decision domain, there may be individual and developmental differences in information‐seeking strategies. During adolescent development, individuals may differ in how they seek information, and in the characteristics they value in their peers. These differences may arise from changes in social goals, changes in strength of peer relationships over time, or increased selectivity as adolescents gain more knowledge about themselves, their peers and the social world in general. Only a few empirical studies have explored how the impact of close others (i.e. friends) and popular peers changes across adolescence. A recent study suggests that as adolescents grow older, they become more sensitive to friends compared to non‐friends (Slagter et al. 2023). However, the effect of age on susceptibility to popular peers remains inconclusive (Field and Prinstein 2023; Pinho et al. 2021; Slagter et al. 2023). Thus, age‐dependency in the importance of specific peer characteristics seems plausible—also beyond closeness and popularity—but remains an open question in the field.

### 1.1 Present Study

Our first aim was to identify characteristics that adolescents use as criteria to select a source of help in a variety of decision domains. Here, we focused on daily life choices within the domain of risky‐, prosocial and academic behaviour. A secondary objective was to investigate whether the characteristics of peers who are consulted are age dependent. To achieve this, we conducted a study with an adolescent sample (*N* = 748, aged 11–19) from preuniversity high schools. Participants were presented with hypothetical daily‐life scenarios and asked to imagine themselves making decisions in these situations. For each scenario, they indicated whether, and which, classmates they would consult before acting. We gathered detailed profiles of each classmate from the participants' perspectives using a peer nomination questionnaire that assessed various characteristics. These nominations were used to predict which peers participants selected as source for behavioural guidance (i.e. social source). We focused primarily on characteristics that describe the closeness of the relationship (e.g., being perceived as a best friend or trustworthy) and the peer's social status (e.g., being perceived as popular or cool). We hypothesised that friendship and trustworthiness would be important characteristics of the selected social sources. Across all domains, we hypothesised that friends would be consulted more frequently than non‐friends. While we expected to find domain‐ and age‐related differences in the characteristics influencing peer selection, we did not have any specific hypotheses regarding the nature of these variations. As such, this study should be considered exploratory, while also serving to test whether previous findings could be replicated.

## 2 Materials and Methods

### 2.1 Participants and Procedure

Data collection took place in classrooms during a regular school hour at Dutch high schools, to target adolescents and their classmates. In the Dutch school system, adolescents transition to high school at around 11 years old. At high school, adolescents follow secondary education in fixed groups of approximately 20–30 students, and these student cohorts remain nearly the same throughout the duration of their secondary education. This fixed group of students spend approximately 26 h per week together in class. We focused on the secondary educational tracks that prepare students for university (6 years) and university of applied sciences (5 years). These tracks have the longest duration in the Netherlands, allowing us to capture a broad age range of students from 11 to 19 years old with a similar peer network structure at school. We included 42 classes from nine Dutch secondary schools, resulting in a total sample of 748 participants (44% female, M age (SD) = 14.6 (1.7)). Participants indicated for a series of described scenarios involving decision‐making in various domains whether they wanted to consult classmates, and if so, which classmates. This data was combined with their response on a peer nomination questionnaire, in which they could indicate which peer characteristics they would attribute to their classmates. These two questionnaires were administered in two separate sessions, with a 2‐week interval between them, as participants took part in a larger study. Peer nominations were obtained 2 weeks before the scenario questionnaire. All participants provided informed consent before data collection. For participants under the age of 16, informed consent was also obtained from their parents or legal guardians. The study procedures were reviewed and approved by the Ethics Review Board of the Faculty of Social and Behavioural Sciences at the University of Amsterdam (case number 2019‐DP‐11427) and were conducted in compliance with relevant guidelines and regulations.

### 2.2 Materials

#### 2.2.1 Peer Nomination Questionnaire

Participants rated all their classmates on various peer characteristics using a peer nomination questionnaire. These characteristics were related to peer status (e.g., “Who is the most popular in class?”), relationship intimacy (e.g., “Who is/are your best friend(s)?”) or behavioural acts (e.g., “Who engages in risky behaviours?”). A complete overview of the peer characteristics can be found in Table 1. We included the peer characteristics from Slagter et al. (2023) to test whether the characteristics identified as being of importance for selecting social sources, would generalise to other behavioural decision domains. For each characteristic, participants could nominate as many classmates as they wished or select the option ‘nobody.’

**Table 1:** List of Nomination Items to Assess the Perceived Characteristics of the Participant's Classmates.

Peer characteristic (Question‐item)
Friend (Who are your friends?)
Best friend (Which classmate(s) is/are your best friends?)
Trustworthy (With whom would you share a secret or your feelings (e.g. that you are in love)
Kind (Which classmates do you like?)
Not kind (Which classmates do you not like?)
Most popular (Which classmates are most popular in your class?)
Influential (Which classmates influence others to do what they want?)
Leader (Which classmates take often the lead in your group/class?)
Smart (which classmates are smart?)
Admirable (Which classmate do you look up to?)
Cool (Which classmates are cool?)
Mean (Which classmates are mean? (e.g. a classmate who bullies excludes or humiliates someone))
Risky (Which classmates do risky things?)

For each participant, we assigned binary codes (1 = attributed, 0 = not attributed) to each classmate based on whether the participant attributed a given characteristic (e.g., “cool”) to that specific classmate. These binary variables, based on the participant's nominations only, were used in subsequent analysis. The associations between peer nominations are disclosed in the supplement (Figure S1), with the strongest association being 0.51. Reciprocal nominations nor group nominations were required for the analysis, as we solely focused on the participant's own perception about their classmates.

#### 2.2.2 Scenarios Questionnaire

Participants were presented with scenarios describing a hypothetical situation in which the participant needed to decide. The scenarios depicted situations that could happen in adolescents' daily lives. The scenarios represented decision‐making within the following domains: risk‐, prosocial behaviour and academia‐related behaviour (see Table S1 for an overview of the scenarios). We focused on these domains because they are key areas of research on peer influence, with studies showing significant peer effects in these behaviours (e.g., Raabe et al. 2019; Shin 2022; Simons‐Morton et al. 2005). Risk scenarios included the following decisions: accepting drugs, getting a vaccination, skipping class or taking alcohol. Prosocial scenarios included the following decisions: taking part in a climate strike, donating to a charity and returning lost money. Academic scenarios included the following decisions: attending a career‐day, signing up for extra classes at school and deciding on which subjects to follow at school. All scenarios described decision‐making within a social context (at a Dutch high school). This made it feasible and realistic to observe the behaviour of other classmates in these scenarios. For each scenario, participants were asked to imagine the situation and think about what they would do. After a time delay of 30 seconds, participants were able to nominate their classmates for the following question: “Whose decision to do [specific to the scenario] would you like to know to help you make a choice?”. Participants could select as many classmates as they wished, or ‘nobody’ if they were not in need for any guidance. The order of scenarios was counterbalanced.

### 2.3 Analytic Plan

We conducted a generalised mixed‐effects model with the selection of classmates as the dependent variable (0 = not selected, 1 = selected). In this model, we included all possible peer characteristics as binary independent variables (see Table 1), and a random intercept at the participant level. To examine whether the characteristics of selected peers were age‐dependent, we included interaction terms between age and each peer characteristic. Age was modelled as a continuous, scaled variable to test for linear age effects. We estimated this main model separately for each domain (i.e., risk, prosocial, and academic behaviour) to identify the types of peers selected as information sources within each domain.

To prevent overfitting and enhance interpretability, we applied a variable selection procedure for each domain‐specific model. We used a generalised mixed‐effects model with LASSO regularisation that forces less informative predictors toward a zero‐coefficient, based on their contribution of explaining the dependent variable and thus their contribution to model fit. This was controlled by a regularisation parameter (λ) that penalises complexity. Higher values of λ result in more coefficients being reduced to zero. See Supplemental Materials, Methods section, for details on selecting a suitable λ value. After variable selection, we re‐estimated a linear mixed‐effects model per domain, including only the meaningful predictors retained by the LASSO regularisation. This allowed us to obtain robust coefficient estimates and rank the remaining predictors by effect size for each domain.

#### 2.3.1 Data Exclusion

Participants that did not consult any peer across all scenario‐items were excluded from the source selection analysis (*N* = 33, 70% Male, M age (SD) = 15.3 (1.8)). See Figure S2 for an age by gender distribution of the analysed and excluded sample). Missing data on item level (i.e. participants skipped answering a scenario) were removed from the analysis, resulting in small sample size differences between the separate domain models (See Supporting Information, Table S2–S4 for the number of observations per model).

## 3 Results

### 3.1 General Information on Adolescents' Peer Consultation for Informed Decision‐Making

Of all participants, 96% (*N* = 715) of the sample preferred to consult a peer at least once. On average, they sought peer input in 19% to 80% of all scenario cases (see Supporting Information Figure S3 for the distribution of consulting behaviour, grouped by age and domain). Participants selected on average two peers for prosocial and risky decisions, and three peers for academic decisions. Detailed frequencies of the number of selected peers by domain and age are provided in Supporting Information Figure S4.

### 3.2 Peer Characteristics Influencing Social Source Selection

**Figure 1:**
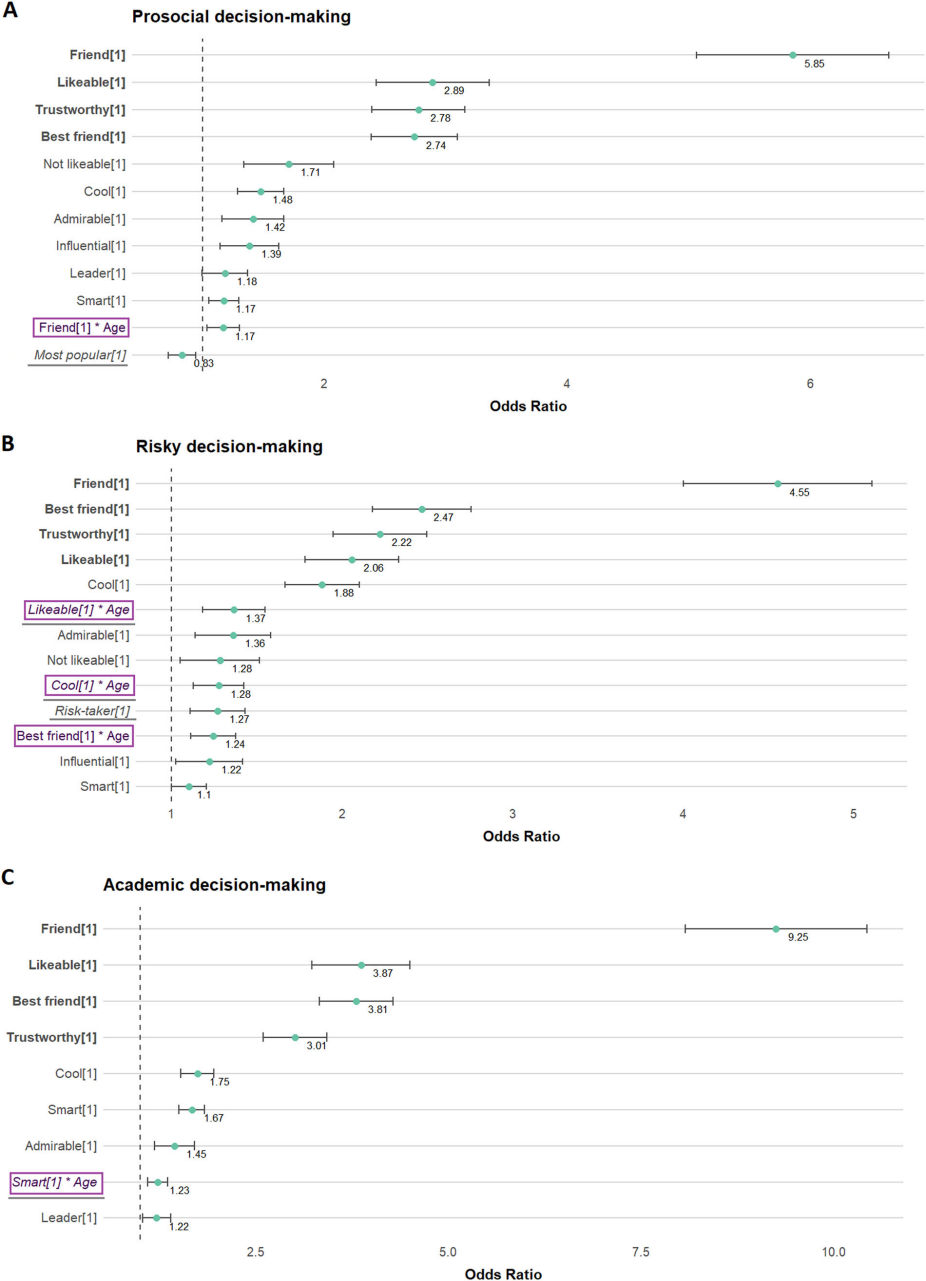
*Characteristics of consulted peers ordered on the odds of being selected. Note:* The most important peer characteristics, which can be seen as criteria for source selection, are depicted for (A) prosocial, (B) risky‐ and (C) school‐related decisions based on their effect size and significance level (*p* < 0.05). Dots represent the odds ratio (OR) and the inverted beta logits, while bars indicate the confidence interval. For all predictive characteristics, estimates above 1 represent an increased chance of being selected as a social source, whereas estimates below 1 represent a decreased chance. Peer characteristics are ordered by effect size from highest to lowest. The top four peer characteristics with an odds ratio above 2 are bolded. Peer characteristics that vary in effect size depending on age are colour‐coded in purple and outlined. Those that suggest domain dependency are underlined and italicised.

We identified several predictive characteristics that contributed uniquely to whether a peer was selected as source, for each decision domain separately (see Supporting Tables S2–S4). All predictors had a variance inflation factor below 2.6, indicating absence of multicollinearity in the final models. Figure 1 highlights, for each domain, the best set of predictors based on their significance level. In this figure, we highlighted the main predictors based on effect size in bold; age‐dependent predictors are colour‐coded in purple and outlined, while domain‐dependent predictors are underlined and italicised. Below we address the predictive characteristics grouped by their commonalities and differences across domains and their age‐dependency. Complete statistical measure outcomes of all domain models can be found in Supporting Tables S2–S4.

#### 3.2.1 Main Predictive Characteristics Across Domains

For all decision domains, we found that friendship status (including its strength), likeability, and trustworthiness were the most important predictors based on their effect size (all Odds ratio (OR) > 2, with *p* < 0.001). In all domains, friendship status showed to be the strongest predictor for selection. Friends had 4.5 to 10 times higher odds of being selected as social source compared to non‐friends, depending on the decision domain. Behaviourally, this was reflected in the high selection rate for friends, with 78.2%, 84.7%, and 70.7% of the peers selected being friends within the prosocial, academic, and risk‐taking domains, respectively.

#### 3.2.2 Additional Predictive Characteristics Across Domains

Peers perceived as being cool, admirable, and/or smart had higher odds of being selected as a social source than peers not nominated for one of these characteristics (OR = 1.1–1.75, all *p* < 0.05).

#### 3.2.3 Domain Variations in Additional Predictive Characteristics

Some predictors were not identified as meaningful across all decision domains, based on their effect size and significance. ‘Not likeable’ peers were more likely to be selected as social sources for prosocial (OR = 1.71, *p* < 0.001) and risky decisions (OR = 1.28, *p* < 0.001), but not for academic decisions. Being perceived as ‘risk‐taker’ was a positive predictor for risky decisions only (OR = 1.27, *p* < 0.001). While the peer's popularity status did not inform peer selection for academic and risky decisions, for prosocial decisions popularity was associated with lower odds of being selected (OR = 0.83, *p* < 0.001). Lastly, depending on the domain, being a ‘leader’ and/or perceived as ‘influential’ predicted peer selection positively (OR = 1.18–1.39, all *p* < 0.05).

#### 3.2.4 Age‐Dependency

The strength of bias for selecting (best) friends showed to vary with age for prosocial and risky decisions. The predictive power of (best) friendship status increased by 1.17 times for prosocial decisions and by 1.24 times for risky decisions with each unit increase in age. For academic decisions, the predictive power of ‘being smart’ increased with age, indicated by the increased odds of selecting smart peers with age (OR = 1.23, *p* < 0.001). For risky decisions, we also found an age‐dependency for likeability and being cool: older adolescents selected more often on these features (OR = 1.28–1.37, all *p* < 0.001). Additionally, a negative main effect of participants' age on peer selection was found across all decision domains, indicating that the odds of selecting a peer as a social source decreased with each unit increase in age (see Supporting Tables S2–S4). We conducted an age sensitivity analysis by excluding low‐frequency age bins (11‐, 18‐, and 19‐year‐olds; see Figure S2A). The results remained consistent (see Supporting Tables S5–S7).

## 4 Discussion

Adolescents often consult or observe their peers before deciding. However, it remains unclear which type of peers adolescents prefer to consult for their daily life choices. This knowledge is important for understanding the peer characteristics contributing to adolescents' openness to peer influence. This study examined which peers adolescents prefer to consult within their school‐based social network across three behavioural decision domains: risk‐taking, prosocial behaviour, and academic behaviour. Across all domains, we found that friendship, being best friends, trustworthiness, and likeability were the four most important selection criteria, with friendship being the strongest predictor. Next to the four main selection criteria, we also identified other criteria (e.g. being cool, admirable, a risk‐taker or smart) that were significant predictors of source selection, however, these varied in strength across the domains. In addition, we identified age‐dependent selection criteria: adolescents' preference for (best) friends increased with age for risky and prosocial decisions, while the preference for smart peers increased with age for academic choices. The discussion is organised around these main findings and further elaborates on the age and domain dependency of these criteria.

### 4.1 Close Friends Can be Seen as the Most Important Social Source

Across all domains, adolescents mainly focus on (best) friends, peers they like and perceive as trustworthy when consulting their social network. These primary selection criteria are consistent with our earlier experimental study focusing on risky decision‐making (Slagter et al. 2023). Friends showed to be the greatest determinant across all domains and ages: friends are 5 to 10 times more likely to be chosen as a social source compared to non‐friends, resulting in friends being selected in 70% to 84% of cases. This bias for friends as sources aligns with existing research on the influence of friends in these behavioural domains (e.g. Burk et al. 2012; Shin 2022; Van Goethem et al. 2014) and enhances our understanding of *how* friends influence others. We contribute to previous literature by demonstrating that friends are also influential because they are actively sought out as a source of help, emphasising adolescents' agency in choosing who influences their behaviour with a preference for friends. For risky and prosocial decisions, where optimal choices depend on subjective preferences and local norms, friends can be seen as valuable sources of information because they likely have similar preferences (Hafen et al. 2011; Laursen 2017). For academic‐related decisions, friends might be a great source of interest, as these academic decisions (i.e. which courses to take) would impact the time adolescents can spend with their best companions at high school. In addition to friendship, we identified ‘kindness’ and ‘trustworthiness’ as key criteria for peer selection. Kind and trustworthy peers were more likely to be chosen as sources to inform decisions. These characteristics also help with differentiating between various type of friends. Not all friends were equally likely to be selected—those viewed as best friends or seen as trustworthy as well were even more likely to be selected. Friends who are considered as trustworthy or best friend may signal high friendship quality. This is in line with previous studies that showed that the magnitude of influence of friends depends on the quality of the relationship and the friend's characteristics (i.e. likeability, Barry and Wentzel 2006; Bot et al. 2005; Laursen et al. 2023; Tucker et al. 2014).

### 4.2 The Social Status of Peers Also Influences Adolescents' Interest in Their Behaviour

Besides the four major predictors mentioned above, we also identified another set of secondary characteristics. That is, we found that classmates perceived as cool, admirable, leaders, smart and/or influential were more likely to be consulted as social sources (see also Slagter et al. 2023). These characteristics help with identifying the other 20%–30% of non‐friend peers that adolescents seek out for guidance but also help with predicting which type of friends are most likely to be consulted. Previous literature addressed these characteristics as indicators of social status (e.g. Cheng 2020; Closson 2009; Lease et al. 2002), suggesting that adolescents with a high social status are more likely to be chosen. At first sight, this seems to contradict our finding that perceived popularity was not used as a selection criterion, and that these peers were even less preferred than others for prosocial decision‐making. However, these findings underscore the importance of distinguishing between the proposed distinct forms of social status, including social preference (being well‐liked and accepted), perceived popularity (Parkhurst and Hopmeyer 1998; van den Berg et al. 2020), and admiration (Shin 2020; Zhang et al. 2018). The absence of ‘perceived popularity’ as a selection criterion may result from the diverse profile of adolescents who are perceived as popular (De Bruyn and Cillessen 2006a; Lease et al. 2002; Postigo‐Zegarra et al. 2021). Peers perceived as popular may exhibit both prosocial and antisocial behaviours, and often gain status through dominance rather than likability or admiration (Cheng et al. 2013; De Bruyn and Cillessen 2006b; van den Berg et al. 2015). Indeed, only a portion of popular peers were seen as admirable or cool in our sample. Our findings align with prior research showing that friends high in social preference or with many friendships, rather than their perceived level of popularity, are more influential than friends with a lower level of social preference (Laursen et al. 2023; Tucker et al. 2014; Zingora et al. 2020). Overall, these findings underscore the importance of considering different dimensions of social status (e.g., popularity, preference, admiration) and highlight the need for clearly defining how social status is operationalized when examining its moderating role in peer influence. How individuals define popular peers—and the traits they associate with them—can differ across age groups and cultural backgrounds (Lansu et al. 2023; Xie et al. 2006; Zhang et al. 2018). To enhance the comparability of findings across age‐groups and cultural backgrounds, it may be more effective to describe influential peers by means of concrete characteristics, obtained with peer nomination assessments.

### 4.3 The Importance of Friends Increases With Age for Risky and Prosocial Decisions

Our study also identified age‐dependency of certain characteristics, indicating that some characteristics become more important as selection criteria. We found that for choices related to risk and prosocial decisions adolescents tend to consult their (best) friends more often with increasing age. Previously, this age effect was also found when investigating adolescent's sampling behaviour for an abstract gambling game (Slagter et al. 2023). The increased focus on friends with age may be the result of increased trust, support, and intimacy that adolescents experience from a longer‐existing friendship (De Goede et al. 2009; Sharabany et al. 1981; Way and Greene 2006). For example, adolescents' perceptions of their friendship quality improve from middle to late adolescence (Way and Greene 2006) and adolescents show increasingly more sharing and trusting behaviours toward their friends with increasing age (Güroglu et al. 2014), resulting in a greater differentiation between friends and other classmates for older adolescents. In addition to the increased quality of the friendship, it is also likely that adolescents will feel more similar to their friends over time (Burk et al. 2012; Gremmen et al. 2017; Shin and Ryan 2014). This could make friends, over the duration of the friendship, an increasingly valuable source to inform your preferences when uncertain (Analytis et al. 2018; Hohman et al. 2014; Moutoussis et al. 2016). Interestingly, for academic choices, we did not observe an increased focus on friends with increasing age, but rather on smart peers, while the overall tendency to consult peers decreased with age. Together, these age‐dependent results support and extend previous findings that friends become an even more important source during adolescence for risky and prosocial (daily‐life) decisions, and suggest that adolescents become more selective in whom to consult, depending on the decision domain.

### 4.4 Domain Variations in Selection: An Adaptive Selection That Grows With Age?

Across all domains, we identified the same set of main selection criteria, with the selection on friends being the most evident. Also, most additional selection criteria were similar, suggesting that source selection within adolescent peer groups is not highly domain‐dependent. However, we did find some domain differences for the additional selection criteria, suggesting that adolescents also considered the appropriateness of the source based on the decision domain. We found that peers perceived as risk‐takers are valued above non‐risk‐takers, but only in the context of risky decision‐making. Here, adolescents might see risk‐takers as valuable sources of information because these peers have direct experience with risk‐taking. Therefore, observing a risk‐taker choosing to refrain from risky behaviour can serve as a stronger indicator of potential danger than if a non‐risk‐taker is refraining from risk‐taking. In addition, we identified age‐depending characteristics that varied by domain: for older adolescents we observed an increased preference for smart peers when facing academic decisions, for cool peers when dealing with risky decisions, and for friends when facing prosocial and risky decisions. Here, smart peers are likely seen as knowledge for academic choices, cool peers as knowledgeable for risky decisions as these peers can show them how to act to increase their own level of ‘coolness’ (Defoe et al. 2022), and friends as knowledgeable for informing them about their own risk and prosocial‐preferences (Analytis et al. 2018). Together, these findings seem to suggest that adolescents also consider the usefulness of the source depending on the situation they face, and this adaptive selection might grow when adolescents get older.

### 4.5 Limitations and Future Directions

Our design allowed us to investigate which peers adolescents prefer to consult within an ecological valid context: we focused on adolescents' own school network when facing daily‐life choices. To serve this ecological strength we also needed to accept some limitations in our current design. Based on this study we cannot draw conclusions about the actual impact of the preferred social sources, nor can we determine the extent to which adolescents benefitted from consulting others. Ethical constraints prevented us from revealing the actual peers' choices, as this is seen as sensitive information, limiting our ability to measure their influence on adolescents' choices. Experimentally, revealing and measuring the impact of familiar peers is only feasible for decisions in abstract tasks that do not include a clear right or wrong answer (i.e. gambling task; Slagter et al. 2023) or norm expectations (i.e. estimation task; Gradassi et al. 2023). Future studies could set‐up a diary study, in which adolescents report about their decision‐making process, including the type of decision, who they consulted and why, and whether the peer choice or advice impacted their decision. Secondly, our study focused on source selection within adolescent's peer network at school. However, adolescents' social network also includes their family, teachers and peers from other communities. Friendship networks may vary based on the ethnicity of the group. For instance, the number of friends adolescents have at school and outside school (e.g., from their neighbourhood or sport club) might differ between ethnic groups (e.g. Barry and Wentzel 2006). To capture the full scope of adolescents' social networks, follow‐up studies could first compile a comprehensive list of sources with whom adolescents interact, both in and outside of school, and include this list of possible sources in the nomination and scenario questionnaire.

Below, we outline key future directions that can expand on our study's design and findings. An interesting next step is to fully understand the (developmental) motives behind when and why adolescents prefer certain social sources over others. Our study set‐up can be extended by including a qualitative part, such as an interview with adolescents, or open‐ended questions, that focuses on investigating adolescents' motivation for each preferred social source. This is especially important when future studies want to consider the appropriateness and adaptiveness of adolescent's source selection. For example, the need for accuracy would point toward a different source (i.e. knowledgeable peer), than the need for social connectiveness (i.e. a friend). Therefore, the appropriateness of preferred social sources should be considered in light of adolescents' motives, next to classifying the consequences in costs or benefits when adhering to the source. Furthermore, future studies should focus on gaining a better understanding of the individual differences in the frequency of consulting peers. One potential factor explaining these individual differences might be prior experience and adolescent's uncertainty about how to behave. Prior experience and uncertainty about how to behave may explain individual differences in adolescents' consulting behaviour. Familiar situations likely boost confidence, while unfamiliar ones may trigger a ‘copy‐when‐uncertain’ strategy (Laland 2004; Kendall et al. 2018). This may also explain why consulting peers decreased with age within our study, as experience with daily‐life situations grows with age. This is supported by Reiter et al. (2021) who showed that uncertainty in choice preference decreases with age, and subsequently, leads to a reduced susceptibility to peer influence. Additionally, future research could focus on the social status and the social goals pursued by the decision‐maker (e.g., seeking intimacy and closeness, prestige, or approval; Ojanen et al. 2005; Ryan and Shim 2006). These factors may help explain individual differences in the preference to consult high‐status peers or friends, or in the preference to consult a peer group as majority versus specific peers, and vice versa. Lastly, the scenario questionnaire can be extended to any other domains of interest, such as decisions related to an adolescent's physical and mental well‐being (i.e. exercising and eating habits; Chung et al. 2017). Additionally, knowledge‐based scenarios (e.g., preparing for a math exam) and peer characteristics focused on specific skills and knowledge can be added to the peer nomination questionnaire, to examine how expertise influences source selection. In all cases, future studies building on this design should involve adolescents, allowing them to contribute with their perspective on relevant decision scenarios and peer characteristics to include.

### 4.6 Implications for Peer Interventions and Education

There is a growing interest in understanding how to use social networks to promote healthy behaviours (e.g. Centola 2011; Dodd et al. 2022; Veenstra and Laninga‐Wijnen 2022). Our study provides some implications for these types of peer interventions. Targeting peers with a high number of friendships within a class or school (Paluck et al. 2016) is likely be more effective for diffusing behaviour than focusing on those perceived as popular. In line with a recent recommendation, adolescents will likely be more open to influence of peers similar to them, such as friends (Centola 2011). This approach becomes particularly important during late adolescence when the reliance on friends seems strongest. However, friend‐led interventions may not always be feasible, as friends often share similar norms and behaviours and may not be willing or able to serve as role models for the desired behaviour. In such cases, other peer characteristics, such as being ‘cool,’ ‘admirable,’ or ‘smart,’ should be considered. Generally, we recommend peer interventions to focus on peers perceived by adolescents as valuable sources of information, as these peers are likely to generate the greatest willingness to follow their behaviour and advice. Our study also has educational implications, as it underscores the importance of treating adolescents as active agents who seek out information from important figures. Educational efforts focused on peer influence have traditionally aimed at making adolescents aware of peer pressure and teaching them how to resist it. However, consulting one's social network can also lead to positive outcomes, making it essential for adolescents to understand their own role in selecting social sources. Encouraging discussions in classrooms about which peers they consult, when, and why will be valuable. To achieve this aim, the scenario questionnaire can be used to help adolescents gain insight into whom they consult and can serve as a starting point for discussion. Engaging adolescents in conversations that stimulate them to reflect on whether they can learn from these sources, will be a productive approach in high school settings.

## 5 Conclusion

Our study reveals that adolescents are inclined to consult their peers when making decisions related to risky behaviour, prosocial behaviour, and academic choices. This study revealed a detailed profile of peers important for behavioural guidance, which appears to be more consistent across domains than highly domain specific. Primarily, adolescents seek information from (best) friends, and peers they like and trust, with friends being the most important source for guidance. For prosocial and risky decisions, the preference for friends becomes even stronger with age. Additionally, peers perceived as cool, admirable, leaders, smart and/or influential were also more likely to be consulted. While friends showed to be very important, these additional characteristics help to refine the profile of consulted friends and non‐friends. Future studies should incorporate adolescents' perspectives through qualitative measures to better understand their motives for choosing certain peers for guidance, and to identify any overlooked characteristics that influence their selection of these peers. In addition, future studies should address to what extent adolescents consider the knowledge and skills of peers when consulting them.

## Conflicts of Interest

The authors declare no conflicts of interest.

## Supporting information

jad70063‐sup‐0001‐Supplemental_Materials_TheSocialSourcesAdolescentsPreferToConsult.

## Data Availability

All data and code supporting the findings of this study are made available on the first author's OSF page: https://osf.io/f3vyn/?view_only=aaa6f23629a840759c0f3b376b5abd8b.
