# Supplementary material for: The Social Sources Adolescents Consult for Daily Life Choices: Variations in Age and Decision Domains
Source: J Adolesc. 2025 Oct 12;98(1):296–306. doi: 10.1002/jad.70063 (PMC12780656; doi:10.1002/jad.70063)
Supplement: Supplementary file 1 — jad70063‐sup‐0001‐Supplemental_Materials_TheSocialSourcesAdolescentsPreferToConsult. [file JAD-98-296-s001.docx]

**Supplementary materials for**

**The Social Sources Adolescents Consult for Daily Life Choices:**

**Variations in Age and Decision Domains**

# **Contents**

1. Supplementary Figures
2. Supplementary Tables
3. Supplementary Methods

#

**Supplementary Figures**

**Figure S1***Associations between peer characteristics based on the full sample (N=715)***
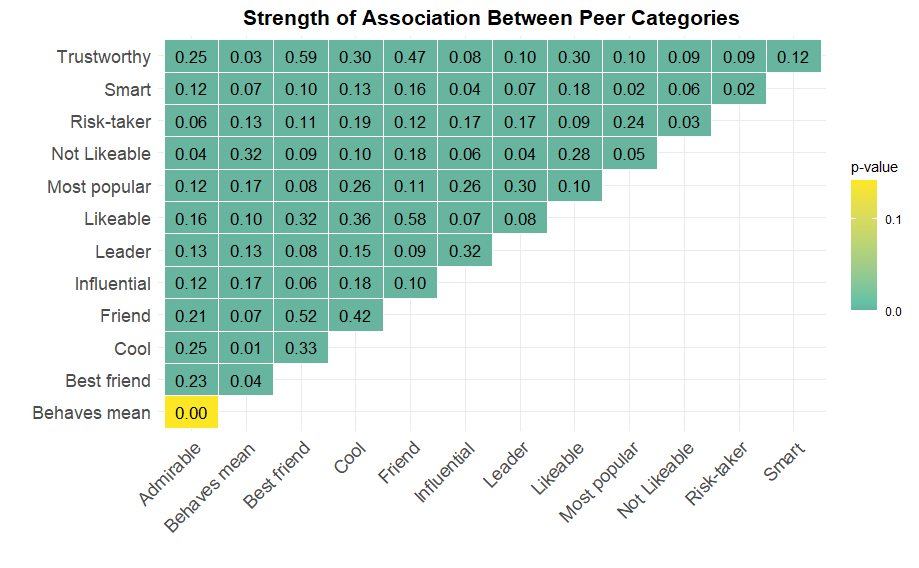
**

**Figure S2***Age and Gender distribution of the analysed sample (N=715) and excluded sample (n=33)*

**
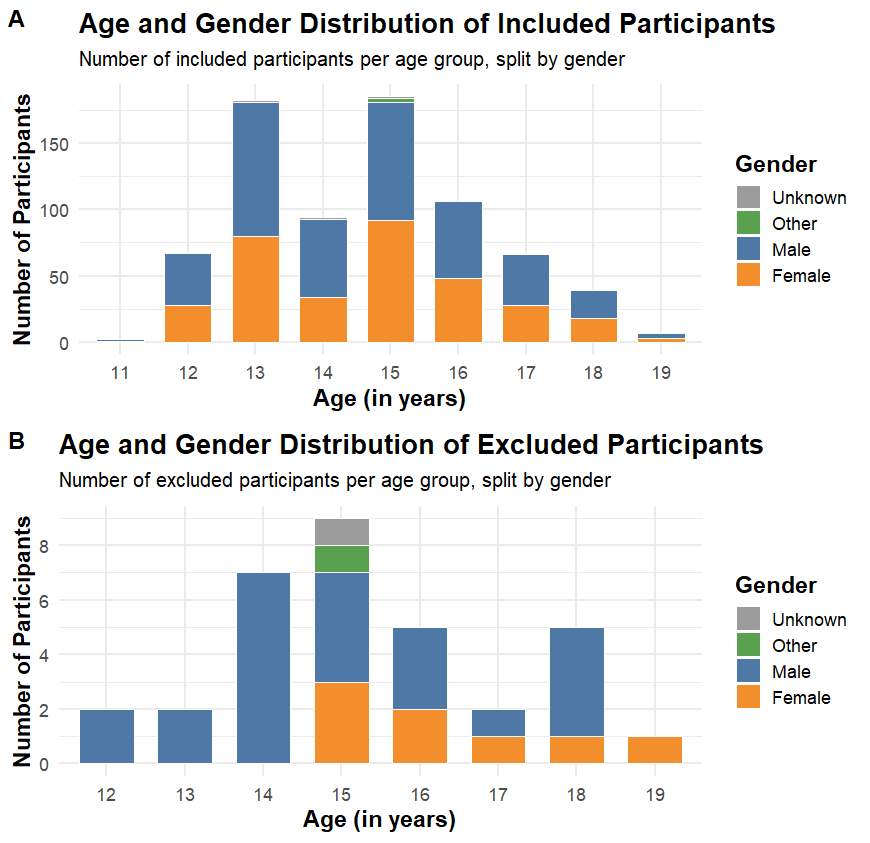
**

**Figure S3***Percentage of scenarios for which participants consulted a peer (N=715)*

**
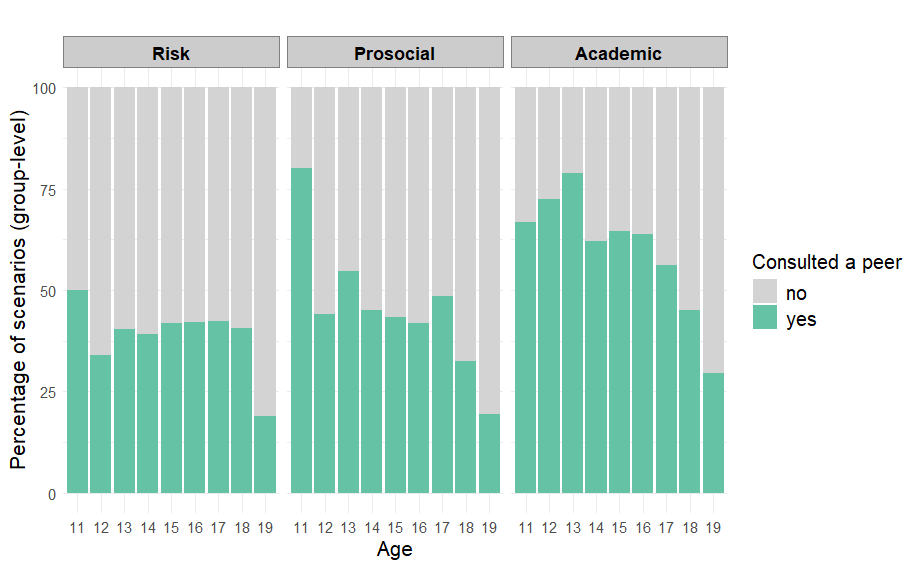
***Note.* Distributions of the percentage of scenarios for which participants decided to consult at least one peer, presented per domain and grouped per age bin. Percentages are based on the group-level mean. These distributions are based on the sample of participants (N=715) used for the main analysis. Participants who did not consult anyone for all scenarios are excluded.

**Figure S4***Distribution of the number of peers selected within each decision domain

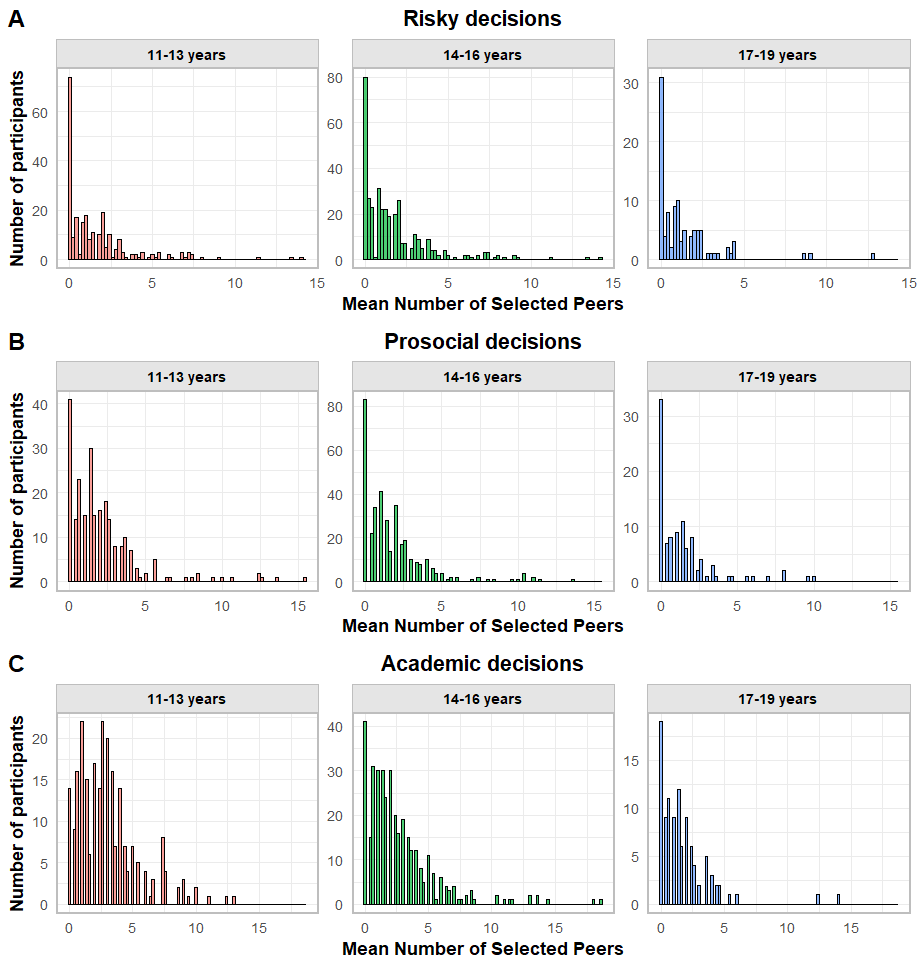
*

*Note.* Distribution of the number of peers selected (mean per participant across the scenarios) per decision domain. These distributions are based on the sample of participants (N=715) used for the main analysis. Participants who did not consult anyone for all scenarios are excluded.

**Supplementary Tables**

**Table S1***Items of the developed hypothetical scenario questionnaire, ordered by behavioural domain.*

| **Domain** | **Scenario topic** | **Scenario description** | **Question of interest that follows the scenario description** | **Answer option(s) that could be selected [unlimited]** |
| --- | --- | --- | --- | --- |
| Risky decisions | Trying drugs | Imagine that at a school party, one of your classmates approaches you and whispers in your ear that you can try drugs in the bathroom. Your classmate can tell you who else is joining. | Whose decision to try drugs would you like to know to help you make your choice? | Names of all their classmates and nobody  (if nobody was selected, other answer options were automatically disabled for selection) |
|  | Skipping class | Imagine you are at school and have one hour left of class. Some classmates decide to skip school. | Whose decision to skip class would you like to know to help you make your choice? | Names of all their classmates and nobody  (if nobody was selected, other answer options were automatically disabled for selection) |
|  | Getting a vaccination | Imagine you are about to receive an invitation to get a (booster) vaccination against a new virus. | Whose decision to get vaccinated would you like to know to help you make your choice? | Names of all their classmates and nobody  (if nobody was selected, other answer options were automatically disabled for selection) |
|  | Taking alcohol | Imagine you are at a party hosted by a classmate. You go to get a drink and see a table with a variety of drinks: soft drinks, as well as beer and wine. | Whose decision to drink alcohol would you like to know to help you make your choice? | Names of all their classmates and nobody  (if nobody was selected, other answer options were automatically disabled for selection) |
| Prosocial decisions | Donating to a Charity | Imagine that a classmate is collecting money for a charity. Your classmate walks by with a list showing which classmates want to donate and the amounts they intend to give. The classmate approaches your table and asks if you would like to donate some of your pocket money to the charity. | Whose decision to donate would you like to know to help you make your choice? | Names of all their classmates and nobody  (if nobody was selected, other answer options were automatically disabled for selection) |
|  | Participate in a climate strike | Imagine that a climate strike is being organized. Your classmates have created a list of participating classmates. A classmate who is joining the strike asks if you would like to join as well. | Whose decision to join the strike would you like to know to help you make your choice? | Names of all their classmates and nobody  (if nobody was selected, other answer options were automatically disabled for selection) |
|  | Found Money | Imagine that you are walking across the schoolyard when an unfamiliar student ahead of you pulls something from their pocket and accidentally drops a 5-euro bill. The student does not notice as you pick up the note, and there are no other people in the schoolyard. | Whose decision on what to do with the money would you want to know to help you make a choice? | Names of all their classmates and nobody  (if nobody was selected, other answer options were automatically disabled for selection) |
| Academia decisions | Visiting a career orientation day | Imagine you are attending a career orientation open day with your school. You can register for various courses and professions to learn more about them. Soon, you will have to choose which courses you want to attend during the open day. | Whose choice of courses and professions would you like to know to help you make a choice? | Names of all their classmates and nobody  (if nobody was selected, other answer options were automatically disabled for selection) |
|  | Choosing your vocational-oriented course package at school | Imagine that you will soon need to choose a study profile at school. This means you must decide which subjects you want to take in the upcoming school year. | Whose choice of courses would you like to know to help you make a choice? | Names of all their classmates and nobody  (if nobody was selected, other answer options were automatically disabled for selection) |
|  | Taking extra lessons for a school subject | Imagine that your school offers the opportunity to attend extra classes. You can choose from any subject, and everyone in your class can choose something different. | Whose choice of subject for extra classes would you like to know to help you make a choice? | Names of all their classmates and nobody  (if nobody was selected, other answer options were automatically disabled for selection) |

*Note*: This table presents the items from the developed hypothetical scenario questionnaire designed to investigate social source selection for guidance in daily life choices. The table includes both the scenario descriptions, clustered by domain, and the corresponding question of interest. Academic scenarios reflect decisions adolescents face while following secondary education in the Dutch school system. For each scenario, participants could select an unlimited number of classmates or indicate that they did not need to consult their peers. The original questionnaire was translated from Dutch to English.

**Table S2***Glmer model output for academic decisions*

|  | **Selecting a peer for advice on academic decisions**  **[1=yes, 0=no]** | | | | |
| --- | --- | --- | --- | --- | --- |
| *Predictors* | *Odds Ratios* | *std. Error* | *CI* | *Statistic* | *p* |
| **(Intercept)** | **0.00** | **0.00** | **0.00 – 0.00** | **-60.07** | **<0.001** |
| **likeable [1]** | **3.87** | **0.32** | **3.28 – 4.56** | **16.16** | **<0.001** |
| not likeable [1] | 1.21 | 0.16 | 0.94 – 1.56 | 1.46 | 0.144 |
| most popular [1] | 0.93 | 0.06 | 0.82 – 1.06 | -1.12 | 0.263 |
| **friendship [1]** | **9.25** | **0.60** | **8.15 – 10.51** | **34.21** | **<0.001** |
| **best friends [1]** | **3.81** | **0.24** | **3.36 – 4.32** | **20.83** | **<0.001** |
| **share secrets [1]** | **3.01** | **0.21** | **2.63 – 3.46** | **15.81** | **<0.001** |
| **look up [1]** | **1.45** | **0.13** | **1.21 – 1.73** | **4.06** | **<0.001** |
| **cool peer [1]** | **1.75** | **0.11** | **1.54 – 1.98** | **8.82** | **<0.001** |
| **smart [1]** | **1.67** | **0.09** | **1.51 – 1.85** | **10.04** | **<0.001** |
| **Leader [1]** | **1.22** | **0.09** | **1.05 – 1.41** | **2.59** | **0.010** |
| Risk-taker [1] | 1.03 | 0.08 | 0.89 – 1.19 | 0.40 | 0.689 |
| **Age scaled** | **0.73** | **0.07** | **0.61 – 0.87** | **-3.51** | **<0.001** |
| likeable [1] × Age scaled | 0.94 | 0.08 | 0.80 – 1.10 | -0.76 | 0.445 |
| friendship [1] × Age scaled | 1.04 | 0.07 | 0.91 – 1.18 | 0.51 | 0.608 |
| best friends [1] × Age scaled | 1.00 | 0.07 | 0.88 – 1.14 | -0.03 | 0.973 |
| share secrets [1] × Age scaled | 1.00 | 0.07 | 0.86 – 1.15 | -0.01 | 0.991 |
| cool peer [1] × Age scaled | 1.01 | 0.06 | 0.90 – 1.15 | 0.20 | 0.840 |
| **smart [1] × Age scaled** | **1.23** | **0.07** | **1.11 – 1.37** | **3.87** | **<0.001** |
| **Random Effects** | | | | | |
| σ^2^ | 3.29 | | | | |
| τ_00_ _id_self_ | 2.55 | | | | |
| ICC | 0.44 | | | | |
| N _id_self_ | 713 | | | | |
| Observations | 57470 | | | | |
| Marginal R^2^ / Conditional R^2^ | 0.413 / 0.670 | | | | |

**Table S3***Glmer model output for risky decisions*

|  | **Selecting a peer for advice on risky decisions  [1=yes, 0=no]** | | | | |
| --- | --- | --- | --- | --- | --- |
| *Predictors* | *Odds Ratios* | *std. Error* | *CI* | *Statistic* | *p* |
| **(Intercept)** | **0.00** | **0.00** | **0.00 – 0.01** | **-61.45** | **<0.001** |
| **likeable [1]** | **2.06** | **0.14** | **1.80 – 2.35** | **10.56** | **<0.001** |
| **not likeable [1]** | **1.28** | **0.12** | **1.07 – 1.54** | **2.71** | **0.007** |
| most popular [1] | 1.04 | 0.06 | 0.92 – 1.17 | 0.60 | 0.551 |
| **friendship [1]** | **4.55** | **0.28** | **4.03 – 5.14** | **24.42** | **<0.001** |
| **best friends [1]** | **2.47** | **0.15** | **2.19 – 2.78** | **15.03** | **<0.001** |
| **share secrets [1]** | **2.22** | **0.14** | **1.96 – 2.51** | **12.63** | **<0.001** |
| **look up [1]** | **1.36** | **0.11** | **1.16 – 1.60** | **3.70** | **<0.001** |
| **cool peer [1]** | **1.88** | **0.11** | **1.68 – 2.11** | **10.75** | **<0.001** |
| **smart [1]** | **1.10** | **0.05** | **1.00 – 1.21** | **2.00** | **0.045** |
| **influential [1]** | **1.22** | **0.10** | **1.04 – 1.43** | **2.44** | **0.015** |
| Leader [1] | 1.06 | 0.08 | 0.92 – 1.23 | 0.79 | 0.428 |
| **Risk-taker [1]** | **1.27** | **0.08** | **1.12 – 1.44** | **3.68** | **<0.001** |
| **Age scaled** | **0.73** | **0.06** | **0.62 – 0.86** | **-3.73** | **<0.001** |
| **likeable [1] × Age scaled** | **1.37** | **0.09** | **1.19 – 1.56** | **4.57** | **<0.001** |
| friendship [1] × Age scaled | 0.90 | 0.06 | 0.79 – 1.02 | -1.58 | 0.114 |
| best friends [1] × Age scaled | 1.24 | 0.07 | 1.12 – 1.38 | 4.04 | **<0.001** |
| cool peer [1] × Age scaled | 1.28 | 0.08 | 1.14 – 1.43 | 4.11 | **<0.001** |
| **Random Effects** | | | | | |
| σ^2^ | 3.29 | | | | |
| τ_00_ _id_self_ | 2.93 | | | | |
| ICC | 0.47 | | | | |
| N _id_self_ | 715 | | | | |
| Observations | 75794 | | | | |
| Marginal R^2^ / Conditional R^2^ | 0.224 / 0.589 | | | | |

**Table 4***Glmer model output for prosocial decisions*

|  | **Selecting a peer for advice on prosocial decisions**  **[1=yes, 0=no]** | | | | |
| --- | --- | --- | --- | --- | --- |
| *Predictors* | *Odds Ratios* | *std. Error* | *CI* | *Statistic* | *p* |
| **(Intercept)** | **0.00** | **0.00** | **0.00 – 0.00** | **-59.21** | **<0.001** |
| **likeable [1]** | **2.89** | **0.24** | **2.46 – 3.40** | **12.94** | **<0.001** |
| **not likeable [1]** | **1.71** | **0.19** | **1.38 – 2.12** | **4.89** | **<0.001** |
| **most popular [1]** | **0.83** | **0.06** | **0.73 – 0.95** | **-2.64** | **0.008** |
| **friendship [1]** | **5.85** | **0.40** | **5.11 – 6.70** | **25.70** | **<0.001** |
| **best friends [1]** | **2.74** | **0.18** | **2.41 – 3.12** | **15.23** | **<0.001** |
| **share secrets [1]** | **2.78** | **0.19** | **2.42 – 3.19** | **14.56** | **<0.001** |
| **look up [1]** | **1.42** | **0.13** | **1.18 – 1.69** | **3.81** | **<0.001** |
| **cool peer [1]** | **1.48** | **0.10** | **1.30 – 1.68** | **6.02** | **<0.001** |
| **smart [1]** | **1.17** | **0.06** | **1.06 – 1.30** | **2.99** | **0.003** |
| **influential [1]** | **1.39** | **0.12** | **1.16 – 1.65** | **3.66** | **<0.001** |
| **leader [1]** | **1.18** | **0.10** | **1.01 – 1.39** | **2.09** | **0.036** |
| Risk-taker [1] | 0.98 | 0.08 | 0.85 – 1.15 | -0.20 | 0.845 |
| **Age scaled** | **0.73** | **0.06** | **0.63 – 0.86** | **-3.95** | **<0.001** |
| **friendship [1] × Age scaled** | **1.17** | **0.07** | **1.04 – 1.31** | **2.69** | **0.007** |
| best friends [1] × Age scaled | 1.08 | 0.07 | 0.94 – 1.23 | 1.08 | 0.280 |
| share secrets [1] × Age scaled | 1.00 | 0.07 | 0.87 – 1.15 | -0.03 | 0.976 |
| **Random Effects** | | | | | |
| σ^2^ | 3.29 | | | | |
| τ_00_ _id_self_ | 2.52 | | | | |
| ICC | 0.43 | | | | |
| N _id_self_ | 710 | | | | |
| Observations | 56865 | | | | |
| Marginal R^2^ / Conditional R^2^ | 0.300 / 0.603 | | | | |

**Table S5***Model output of the age sensitivity analysis for academic decisions (excluding 11-,18- and 19-year-olds)*

|  | **Selecting a peer for advice on academic decisions [1=yes, 0=no]** | | | | |
| --- | --- | --- | --- | --- | --- |
| *Predictors* | *Odds Ratios* | *std. Error* | *CI* | *Statistic* | *p* |
| **(Intercept)** | **0.00** | **0.00** | **0.00 – 0.00** | **-58.11** | **<0.001** |
| **likeable [1]** | **3.83** | **0.33** | **3.24 – 4.54** | **15.59** | **<0.001** |
| **not likeable [1]** | **1.24** | **0.16** | **0.96 – 1.60** | **1.62** | **0.106** |
| **most popular [1]** | **0.94** | **0.06** | **0.82 – 1.07** | **-0.97** | **0.331** |
| **friendship [1]** | **9.46** | **0.63** | **8.31 – 10.78** | **33.82** | **<0.001** |
| **best friends [1]** | **3.90** | **0.25** | **3.44 – 4.44** | **20.92** | **<0.001** |
| **share secrets [1]** | **2.98** | **0.21** | **2.59 – 3.42** | **15.43** | **<0.001** |
| **look up [1]** | **1.41** | **0.13** | **1.18 – 1.69** | **3.70** | **<0.001** |
| **cool peer [1]** | **1.75** | **0.11** | **1.55 – 1.99** | **8.74** | **<0.001** |
| **smart [1]** | **1.66** | **0.09** | **1.50 – 1.84** | **9.81** | **<0.001** |
| **lead [1]** | **1.20** | **0.09** | **1.03 – 1.40** | **2.39** | **0.017** |
| Risk-taker [1] | 1.02 | 0.08 | 0.87 – 1.18 | 0.20 | 0.840 |
| **Age scaled** | **0.82** | **0.08** | **0.68 – 0.98** | **-2.13** | **0.033** |
| likeable [1] × Age scaled | 0.87 | 0.07 | 0.74 – 1.02 | -1.71 | 0.088 |
| friendship [1] × Age scaled | 1.05 | 0.07 | 0.92 – 1.21 | 0.76 | 0.445 |
| best friends [1] × Age scaled | 1.03 | 0.07 | 0.91 – 1.17 | 0.49 | 0.626 |
| share secrets [1] × Age scaled | 0.95 | 0.07 | 0.82 – 1.10 | -0.68 | 0.496 |
| cool peer [1] × Age scaled | 1.03 | 0.06 | 0.91 – 1.16 | 0.46 | 0.646 |
| **smart [1] × Age scaled** | **1.18** | **0.06** | **1.06 – 1.31** | **3.07** | **0.002** |
| Random Effects | | | | | |
| σ2 | 3.29 | | | | |
| τ00 id_self | 2.55 | | | | |
| ICC | 0.44 | | | | |
| N id_self | 671 | | | | |
| Observations | 53150 | | | | |
| Marginal R2 / Conditional R2 | 0.414 / 0.670 | | | | |

***Table S6*** *Model output of the age sensitivity analysis for risky decisions (excluding 11-,18- and 19-year-olds)*

|  | **Selecting a peer for advice on risky decisions [1=yes, 0=no]** | | | | |
| --- | --- | --- | --- | --- | --- |
| Predictors | Odds Ratios | std. Error | CI | Statistic | p |
| **(Intercept)** | **0.00** | **0.00** | **0.00 – 0.01** | **-57.79** | **<0.001** |
| **likeable [1]** | **1.98** | **0.14** | **1.72 – 2.27** | **9.56** | **<0.001** |
| **not likeable [1]** | **1.29** | **0.12** | **1.07 – 1.55** | **2.71** | **0.007** |
| most popular [1] | 1.05 | 0.06 | 0.93 – 1.18 | 0.75 | 0.451 |
| **friendship [1]** | **4.52** | **0.29** | **3.98 – 5.13** | **23.21** | **<0.001** |
| **best friends [1]** | **2.46** | **0.16** | **2.17 – 2.78** | **14.28** | **<0.001** |
| **share secrets [1]** | **2.32** | **0.15** | **2.04 – 2.63** | **12.96** | **<0.001** |
| **look up [1]** | **1.34** | **0.11** | **1.13 – 1.58** | **3.43** | **0.001** |
| **cool peer [1]** | **1.85** | **0.11** | **1.64 – 2.08** | **9.89** | **<0.001** |
| **smart [1]** | **1.11** | **0.05** | **1.01 – 1.22** | **2.12** | **0.034** |
| **influential [1]** | **1.18** | **0.10** | **1.00 – 1.39** | **1.96** | **0.050** |
| leader [1] | 1.06 | 0.08 | 0.92 – 1.23 | 0.80 | 0.424 |
| **Risk-taker [1]** | **1.26** | **0.08** | **1.10 – 1.43** | **3.45** | **0.001** |
| **Age (scaled)** | **0.80** | **0.08** | **0.66 – 0.97** | **-2.23** | **0.026** |
| **likeable [1] × Age scaled** | **1.30** | **0.10** | **1.11 – 1.52** | **3.26** | **0.001** |
| friendship [1] × Age scaled | 0.88 | 0.07 | 0.76 – 1.03 | -1.62 | 0.104 |
| **best friends [1] × Age scaled** | **1.24** | **0.08** | **1.09 – 1.40** | **3.36** | **0.001** |
| **cool peer [1] × Age scaled** | **1.22** | **0.09** | **1.07 – 1.40** | **2.88** | **0.004** |
| Random Effects | | | | | |
| σ2 | 3.29 | | | | |
| τ00 id_self | 3.00 | | | | |
| ICC | 0.48 | | | | |
| N id_self | 673 | | | | |
| Observations | 70285 | | | | |
| Marginal R2 / Conditional R2 | 0.213 / 0.589 | | | | |

***Table S7*** *Model output of the age sensitivity analysis for prosocial decisions (excluding 11-,18- and 19-year-olds)*

|  | **Selecting a peer for advice on prosocial decisions [1=yes, 0=no]** | | | | |
| --- | --- | --- | --- | --- | --- |
| Predictors | Odds Ratios | std. Error | CI | Statistic | p |
| **(Intercept)** | **0.00** | **0.00** | **0.00 – 0.00** | **-59.17** | **<0.001** |
| **likeable [1]** | **2.87** | **0.24** | **2.44 – 3.37** | **12.76** | **<0.001** |
| **not likeable [1]** | **1.73** | **0.19** | **1.39 – 2.15** | **4.98** | **<0.001** |
| **most popular [1]** | **0.83** | **0.06** | **0.72 – 0.95** | **-2.68** | **0.007** |
| **friendship [1]** | **5.90** | **0.41** | **5.15 – 6.75** | **25.74** | **<0.001** |
| **best friends [1]** | **2.74** | **0.18** | **2.41 – 3.12** | **15.22** | **<0.001** |
| **share secrets [1]** | **2.79** | **0.20** | **2.43 – 3.20** | **14.60** | **<0.001** |
| **look up [1]** | **1.39** | **0.13** | **1.16 – 1.67** | **3.62** | **<0.001** |
| **cool peer [1]** | **1.48** | **0.10** | **1.30 – 1.68** | **6.04** | **<0.001** |
| **smart [1]** | **1.19** | **0.06** | **1.07 – 1.32** | **3.22** | **0.001** |
| **influential [1]** | **1.39** | **0.12** | **1.17 – 1.66** | **3.71** | **<0.001** |
| **leader [1]** | **1.18** | **0.10** | **1.01 – 1.39** | **2.06** | **0.039** |
| Risk-taker [1] | 0.98 | 0.08 | 0.84 – 1.14 | -0.31 | 0.754 |
| **Age (scaled)** | **0.77** | **0.06** | **0.66 – 0.90** | **-3.30** | **0.001** |
| **friendship [1] × Age (scaled)** | **1.15** | **0.07** | **1.02 – 1.30** | **2.36** | **0.018** |
| best friends [1] × Age (scaled) | 1.08 | 0.07 | 0.94 – 1.23 | 1.08 | 0.281 |
| share secrets [1] × Age (scaled) | 0.99 | 0.07 | 0.86 – 1.14 | -0.14 | 0.888 |
| Random Effects | | | | | |
| σ2 | 3.29 | | | | |
| τ00 id_self | 2.49 | | | | |
| ICC | 0.43 | | | | |
| N id_self | 706 | | | | |
| Observations | 56360 | | | | |
| Marginal R2 / Conditional R2 | 0.298 / 0.600 | | | | |

**Supplementary Methods**

**Variable Selection Procedure: Determining the Penalization Parameter (𝜆)**

***Determining the optimal value range for the penalization parameter:***We started by testing a broad sequence of lambda (𝜆) values and assessed the AIC values to identify a meaningful range for penalization. The optimal lambda range was determined by fitting a set of values, starting with a high lambda value that would shrink all meaningful predictors to zero, ensuring the sequence began with a value that resulted in a null model. For the risk and prosocial domain models, the tested lambda values ranged from 6000 to 0, and for the academic domain model, from 8000 to 0 (with 𝜆 = 0, meaning no penalization). The models were tested in iterations, with decreasing lambda values in steps of -200.

***Running models with the optimal range for the penalization parameter***:
Based on the AIC values from the initial model runs, we refined the optimal lambda range by visually inspecting the change in AIC values as a function of lambda. We selected a range of lambda values that significantly affected the AIC, eliminating those that no longer impacted the model fit. Models were then tested in steps of -20 within this range. The lambda values tested ranged from 1400-200, 1400-400, and 2400-600 for the risk, prosocial, and academic domain models, respectively.

***Final Model Selection:***
The final lambda value was chosen based on the model with the lowest AIC. This lambda value was used in the lasso regularization model, to obtain the subset of variables that resulted in the best fit based on AIC. Variables not shrunk to zero were considered meaningful predictors and were entered into a general linear mixed model to report their effect size and statistical significance (p-value).
